# Coumaphos Exposure and Incident Cancer among Male Participants in the Agricultural Health Study (AHS)

**DOI:** 10.1289/ehp.0800446

**Published:** 2009-09-11

**Authors:** Carol H. Christensen, Elizabeth A. Platz, Gabriella Andreotti, Aaron Blair, Jane A. Hoppin, Stella Koutros, Charles F. Lynch, Dale P. Sandler, Michael C.R. Alavanja

**Affiliations:** 1 Toxicology and Epidemiology Branch, Health Effects Division, Office of Pesticide Programs, U.S. Environmental Protection Agency, Washington, DC, USA;; 2 Department of Epidemiology, Johns Hopkins Bloomberg School of Public Health, Baltimore, Maryland, USA;; 3 Occupational and Environmental Epidemiology Branch, Division of Cancer Epidemiology and Genetics, National Cancer Institute, National Institutes of Health, U.S. Department of Health and Human Services, Rockville, Maryland, USA;; 4 Epidemiology Branch, National Institute of Environmental Health Sciences, National Institutes of Health, U.S. Department of Health and Human Services, Research Triangle Park, North Carolina, USA;; 5 Department of Epidemiology, University of Iowa, Iowa City, Iowa, USA

**Keywords:** agriculture, cancer, coumaphos, insecticide, livestock, neoplasms, occupational exposure, organophosphate, pesticide, prostate

## Abstract

**Background:**

Coumaphos is an organophosphate livestock insecticide. Previous research in the Agricultural Health Study (AHS) cohort observed a positive association between coumaphos and prostate cancer in men with a family history of prostate cancer.

**Objectives:**

This study was performed to determine the association between coumaphos and other major cancer sites and to explore the consistency of the association with prostate cancer early (1993–1999) and later (2000–2005) in AHS follow-up.

**Methods:**

This study included 47,822 male licensed pesticide applicators. Incident cases were ascertained by linkage to state cancer registries, and exposure data were collected by enrollment questionnaire. Poisson regression was used to estimate rate ratio (RR) and 95% confidence interval (CI) of cancer for coumaphos exposure controlling for potentially confounding variables.

**Results:**

Approximately 8% of applicators reported use of coumaphos; 8.5% reported a family history of prostate cancer. Cumulative exposure to coumaphos was not associated with cancer risk overall or with any major cancer site including prostate. In men with a family history of prostate cancer, we observed a positive association between ever use of coumaphos and prostate cancer in both early (RR *=* 2.07; 95% CI, 1.19–3.62, *p*-interaction = 0.005) and later (RR = 1.46; 95% CI, 0.89–2.40; *p*-interaction = 0.11) periods of follow-up. Across all years, this association was statistically significant (RR = 1.65; 95% CI, 1.13–2.38; *p*-interaction = 0.004).

**Conclusion:**

Coumaphos was not associated with any cancer evaluated here. In men with a family history of disease, there was evidence of an association between coumaphos and prostate cancer, possibly due to genetic susceptibility; however, other explanations, including chance, are plausible.

Coumaphos [*O*,*O*-diethyl *O*-(3-chloro-4-methyl-2 oxo-2H-1-benzopyran-7-*yl*) phosphorothioate], an organophosphate (OP) insecticide first registered in 1958, is used to control pests on beef cattle, dairy cows, goats, horses, sheep, and swine [U.S. Environmental Protection Agency (EPA) 1996]. Approximately 83% of the total chemical usage is on beef and dairy cattle. From 1990 to 1999, approximately 71,000 pounds of coumaphos were used on 6 million livestock annually (Smearman 2000). Coumaphos is applied primarily as a liquid (animal immersed in an insecticide-containing dip) but is also applied through a hand-held sprayer or dust formulation (U.S. EPA 1996). In addition to direct exposure to coumaphos among agricultural workers, family members of agricultural workers may be secondarily exposed in the home through contact with contaminated clothing or equipment (Arcury et al. 2007). The general population may be exposed through consumption of coumaphos-treated food products—for example, milk from coumaphos-treated dairy cattle (Cardeal Zde and Dias Paes 2006). However, commercial pesticide products containing the active ingredient coumaphos cannot be purchased by the general public.

*In vitro* studies indicate coumaphos is not mutagenic using either *Salmonella typhimurium* or *Escherichia coli* assays with and without metabolic activation (U.S. EPA 1996). There was no evidence of carcinogenicity or increased tumor formation in two 2-year feeding studies in different strains of rat (dose range: 1–25 ppm and 10 and 20 ppm, respectively), in one strain of mouse (dose range: 10 and 20 ppm), or in one 1-year feeding study in beagle dogs (dose range: 1–90 ppm) (U.S. EPA 1996). Dosing regimens in the four animal feeding studies were adequate to detect cancer based on range-finding studies. In 1994, the U.S. EPA classified coumaphos as a group E carcinogen: no evidence of carcinogenicity based on studies of at least two animal species. The U.S. EPA reaffirmed the decision in the year 2000 (U.S. EPA 2000). However, the relevant exposure to humans is the commercial product, which contains a mixture of active and inactive (inert) ingredients.

A few epidemiologic studies have associated coumaphos exposure with cancer. Among adult male farmers, the risk of glioma was significantly increased for those who reported ever use of coumaphos [adjusted odds ratio (OR) = 5.9; 95% confidence interval (CI), 1.1–32.0)] (Lee et al. 2005). In a pooled analysis from three case–control studies on multiple pesticide exposures and non-Hodgkin lymphoma (NHL) in midwestern states, the adjusted OR for NHL among those reporting use of coumaphos was 2.4 (95% CI, 1.0–5.8) (De Roos et al. 2003). In a nested case–control study within the Agricultural Health Study (AHS), researchers observed that among those with a family history of prostate cancer, those exposed to coumaphos had a 2-fold increased risk of prostate cancer (OR = 2.17; 95% CI, 1.24–3.82) compared with never users, although there was no increased risk among coumaphos users without a family history (Alavanja et al. 2003). Furthermore, although none of the 10 OP pesticides included in the AHS have been associated with prostate cancer in reports published to date, AHS researchers did observe a significant OR for the interaction [the interaction odds ratio (IOR), the OR for the cross-product term] between family history of prostate cancer and ever use of certain OP pesticides with prostate cancer, including chlorpyrifos (IOR = 1.65; 95% CI, 1.02–2.66, *p =* 0.04), phorate (IOR = 1.64; 95% CI, 1.02–2.63, *p =* 0.04), fonofos (IOR = 2.04; 95% CI, 1.21–3.44, *p =* 0.008), and coumaphos (IOR = 2.58; 95% CI, 1.29–5.18, *p =* 0.008) (Alavanja et al. 2003). The purpose of the present analysis was to evaluate prospectively the association between coumaphos exposure and risk of total and site-specific cancers among men in the AHS across 12 years of follow-up, as well as to examine the consistency later in follow-up (2000–2005) of the previous finding (1993–1999) of a higher risk of prostate cancer in men with coumaphos exposure but only if they have a family history of prostate cancer.

## Material and Methods

### Study population

The AHS is prospective cohort study comprising 57,310 pesticide applicators in Iowa and North Carolina (Alavanja et al. 1996). Pesticide applicators were enrolled in the study between December 1993 and December 1997 and on enrollment were asked to complete a self-administered written questionnaire that solicited information on use of pesticides as well as lifestyle factors (Alavanja et al. 1996). More than 82% of private pesticide applicators in Iowa and North Carolina participated in the study. Records of cohort members were matched to Iowa and North Carolina state cancer registries to obtain information on cancer type, stage, and grade at diagnosis, and with the state death registries and the National Death Index to collect mortality information. This study includes all incident cases from enrollment through 31 December 2005 (AHS, Data Release, version P1REL0612; unpublished data). Residential location was identified through the federal Internal Revenue Service address file, state motor vehicle registration records, recent telephone or mail contact with members, and the restricted-use pesticide license and certification records of the departments of agriculture in Iowa and North Carolina. Study participants were censored for loss to follow-up, exit from the state of enrollment, receipt of a cancer diagnosis, or death; less than 2*%* of the cohort is lost to cancer incidence follow-up. All participants provided informed consent, and the research protocol was approved by all appropriate institutional review boards.

### Exposure assessment

Coumaphos exposure information was collected through the enrollment questionnaire; questionnaires are available online (AHS 2008). Use of coumaphos was characterized using three different exposure metrics: ever/never use of the chemical, lifetime exposure days, and intensity-weighted lifetime exposure days. The lifetime exposure days metric was calculated as the product of the total number of years of coumaphos application and the number of days per year of coumaphos application. The intensity-weighted lifetime exposure day metric incorporates lifetime exposure days weighted by factors that may modify pesticide exposure, including application methods, mixing and equipment repair status, and use of personal protective equipment [(application method + mixing status + equipment repair status) × (use of personal protective equipment)] (Dosemeci et al. 2002). Weights for these factors were based on monitoring data available in the published literature.

### Statistical analysis

From the AHS population, female applicators (*n =* 1,563) were excluded, as there were too few exposed cases to perform an analyses (only one coumaphos-exposed female applicator). To ensure that exposure preceded the development of cancer, we excluded prevalent cancer cases (*n =* 1,063). We also excluded participants who *a*) did not contribute person-years to the cohort (*n =* 10), *b*) did not provide birth date information (*n =* 2), *c*) were enrolled into the study as out-of-state residents and continue to reside out of state (*n =* 285), or *d*) did not provide information on use of coumaphos (*n =* 6,565). After exclusions, 47,822 participants contributing 480,514 person-years at risk were included in this analysis. For the analysis of potential effect modification by presence of family history by time period of prostate cancer diagnosis, there were 217,047 person-years accrued by persons at risk for prostate cancer in the early period and 263,467 person-years of exposure accrued in the later period.

Associations between coumaphos and cancer risk were assessed by calculating incidence rate ratios (RRs) and 95% CIs using Poisson regression. We evaluated all cancers combined, prostate, lung, colorectal, and lymphohematopoietic cancers (leukemia, NHL, multiple myeloma, and Hodgkin lymphoma), because there were five or more cancer cases per exposure category for these cancer sites. Lifetime exposure days and intensity-weighted exposure day metrics were categorized into tertiles based on the distribution among all cancer cases exposed to coumaphos. Cut points were based on all cancer cases, rather than on the baseline cohort, to assure adequate numbers of cancer cases observed per tertile of exposure to perform a statistically robust analysis. To evaluate the possibility of residual (unidentified) confounding between those who never and ever used coumaphos, we performed all analyses using two referent groups: men not exposed to coumaphos and men with low exposure to coumaphos (bottom tertile). We did not statistically adjust for multiple comparisons.

We performed Poisson regression analyses adjusting for age (20–29, 30–39, 40–49, 50–59, ≥ 60 years), education (high school education or less and greater than high school), state of residence, smoking (never, former, current), first-degree family history of organ-specific cancers, fruit and vegetable consumption (two or fewer per week, three to six per week, or seven or more per week), and total days of application of any pesticide. Further adjustment for alcohol consumption, race/ethnicity, enrollment year, use of other correlated pesticides, cattle farming, and other suspected cancer risk factors had minimal influence on the point estimates and were not included in the final model. We analyzed dose–response trends for coumaphos exposure and cancer risk by including the midpoint of each exposure tertile as a continuous variable and testing for the statistical significance of the slope.

Our secondary aim was to determine whether the observed association between coumaphos exposure and incident prostate cancer among those with a family history of prostate cancer in the AHS cohort between enrollment and December 1999 (Alavanja et al. 2003) was also observed among men diagnosed in the later period of follow-up. We evaluated prostate cancer risk in association with ever/never use of coumaphos, stratifying by presence of family history of prostate cancer for both the early period of follow-up (1993–1999) and the later period of follow-up (2000–2005) and then across the entire period of follow-up, adjusting for age. We also investigated evidence of this potential effect modification using categories of lifetime exposure days across the entire follow-up period. We evaluated interaction by generating a cross-product term between coumaphos use and family history that was included in a multivariable model of prostate cancer; we quantified the significance of the interaction term using the Wald test (Rosner 1995). We evaluated whether the previously observed association was homogeneous by category of stage (categorized as local or as regional or distant) and histologic grade (categorized as well differentiated or moderately well differentiated or as poorly differentiated or undifferentiated) of prostate tumor at diagnosis. We also evaluated potential effect modification by family history of all cancer and for organ-specific cancers over the entire follow-up period. All tests for significance were two-sided with α = 0.05. Stata statistical software (release 8.0; StataCorp., College Station, TX) was used for all analyses.

## Results

We observed 2,960 cancer cases, 1,196 of which were prostate, among the 44,133 men who reported no use of coumaphos, and we observed 258 cancers cases, 115 of which were prostate, among 3,689 men who reported using coumaphos. Among the 1,311 prostate cancers, 504 occurred early in follow-up (1993–1999), and 807 occurred later in follow-up (2000–2005). More than one-half of the prostate cancers identified were local stage (55%), 13% were regional or distant, and 32% were not classified as to stage at diagnosis, possibly because the “watchful waiting” treatment strategy was recommended for some cases. Most prostate tumors diagnosed in the cohort were moderately well or well differentiated (70%), 26% were poorly differentiated, 0.3% of tumors were undifferentiated, and the remaining 3.3% of tumors were not determined as to histologic grade. Thirty-eight percent of the cohort reported a family history of any cancer.

At enrollment, 7.7% of the study population reported using coumaphos, and the range of annual exposure days reported by coumaphos users was between 2.5 and 200 days per year. Table 1 displays the frequency of selected demographic characteristics of the study population by categories of lifetime exposure days of coumaphos use. The participants were predominantly non-Hispanic white (97%) male private pesticide applicators. Compared with the low- and high-exposure groups, the unexposed group was generally younger and included a higher proportion of commercial applicator and nonwhites. Men in the unexposed group were less likely to have attained education beyond high school or to report a first-degree family member with a cancer diagnosis (Table 1). The mean (± SD) number of coumaphos exposure days over the lifetime was 7.5 ± 2.5 among men in the lowest exposure group and 167.1 ± 402.1 among men in the highest exposure group. Those with a family history of prostate cancer had twice the risk of prostate cancer (OR = 2.02; 95% CI, 1.73–2.37) (data not shown).

RRs and 95% CIs for selected cancers by categories of coumaphos lifetime exposure days are shown in Table 2. Risk estimates for all cancers combined were not significantly different from the null, regardless of whether the reference group was men never exposed to coumaphos or men with low exposure. No cancer site displayed evidence of an exposure–response gradient. Coumaphos lifetime exposure days were not related to the risk of other major cancer sites examined including prostate overall or when stratified by stage and grade of disease. Results were similar for analyses based on intensity-weighted lifetime exposure and are not shown here.

Table 3 shows the association between coumaphos exposure and prostate cancer risk among men with and without a family history of prostate cancer. Results are presented for later in follow-up (2000–2005) and across the entire follow-up period by ever/never use of coumaphos. For comparison, results are also presented for early in follow-up (1993–1999), which was reported previously (Alavanja et al. 2003). At baseline, approximately 8% of men not exposed to coumaphos reported a family history of prostate cancer, whereas 11% of coumaphos-exposed men reported a family history of prostate cancer. Among men without a family history of prostate cancer, there was no association between coumaphos and prostate cancer early, later, or across the entire follow-up period. However, as observed early in follow-up, among men with a family history of prostate cancer, men who ever used coumaphos had a nonsignificantly higher risk of prostate cancer in follow-up years 2000–2005 than men who did not use the chemical (RR = 1.46; 95% CI, 0.89–2.40). Across the entire 12 years of follow-up, the RR of prostate cancer comparing ever versus never users among those with a family history of prostate cancer was statistically significant (RR = 1.65; 95% CI, 1.13–2.38), as was the test for interaction between coumaphos exposure and family history (*p*-interaction = 0.004).

In a joint-effects analysis of the effect of both a positive family history of prostate cancer and self-reported ever use of coumaphos in association with prostate cancer (age-adjusted only), we observed the joint effect of both exposures in excess of the expected joint effect of the two independent exposures, measured on the multiplicative scale. Compared with the referent group of no reported family history of prostate cancer and no self-reported use of coumaphos, those who report use of the chemical but no family history have no increased risk of prostate cancer (RR = 0.86; 95% CI, 0.69–1.10); those who report a family history but no use of coumaphos have an almost 2-fold increased risk of prostate cancer (RR = 1.75; 95% CI, 1.49–2.05), and those who report both a positive family history of prostate cancer and self-report use of coumaphos have a nearly 3-fold increased risk of prostate cancer (RR = 2.89; 95% CI, 2.04–4.09) compared with the expected joint effect of 1.5. Therefore, those who report both exposures have a nearly 2-fold increased risk beyond the expected joint effect of the two exposure factors if they were independent. Results of the joint effect model are similar in both periods of prostate cancer diagnosis—that is, there is statistically significant effect modification of prostate cancer risk in both the later and earlier follow-up periods.

We observed similar patterns of effect modification by family history of prostate cancer in the relation between lifetime exposure days of coumaphos use and prostate cancer across the entire follow-up period (*p*-interaction = 0.02) (data not shown). Adjusting for age and using measures of cumulative exposure collected at enrollment, we observed increased risk when comparing the highest exposure group (i.e., upper half of exposed group) (*n =* 16 prostate cancer cases) with the nonexposed group (*n =* 184 prostate cancer cases) among men with a family history of disease (RR = 1.36; 95% CI, 0.81–2.26, *p* for trend = 0.04) and observed no increased risk between the top tertile (*n =* 37 prostate cancer cases) and the nonexposed group (*n =* 935 prostate cancer cases) among men without a family history of disease (RR = 0.78; 95% CI, 0.56–1.08, *p* for trend = 0.15). However, the risk of prostate cancer by lifetime exposure day category for men with a family history of disease was not monotonic. There were insufficient data available to investigate the exposure–response relation between coumaphos exposure and prostate cancer risk by presence of family history (yes/no) and by time period of follow-up (early and later periods).

## Discussion

To our knowledge, this is the largest examination of any group occupationally exposed to coumaphos. We observed no overall association between reported use of coumaphos and risk of total cancer or cancers of the prostate, lung, or lymphohematopoietic system, or colorectal cancer. The lack of observed association between coumaphos exposure and all lymphohematopoietic cancers combined may be explained by the low percentage of coumaphos-exposed lymphohematopoietic cancers as well as the known heterogeneity among lymphohematopoietic cancers. We had no *a priori* hypotheses regarding the potential association between coumaphos use and lung cancer or colorectal cancer. Similar to an earlier AHS report, we observed a positive association between ever use of coumaphos and incident prostate cancer among those with a family history of prostate cancer, but not among those without a family history of prostate cancer, in a later follow-up period (2000–2005) as well as across all years of follow-up (1993–2005). However, the observed association was stronger in the early period than in the later period of follow-up. The current study includes more than twice as many prostate cancer cases and 6 additional years of follow-up than the earlier report (Alavanja et al. 2003). Further analysis suggests that the differences between early and later time periods may be due in part to a difference in the number of lifetime days of exposure. Using lifetime exposure days measured at the beginning of follow-up, coumaphos-exposed prostate cancer cases with a family history of disease in the early period reported an average of 130 lifetime exposure days, whereas coumaphos-exposed cases with a family history of prostate cancer in the later period reported an average of 40 lifetime exposure days at baseline. Therefore, the attenuation in risk observed in the later time period of prostate cancer diagnosis may be attributable to lower cumulative exposure. However, we acknowledge that other explanations are possible. The use of exposure information collected at enrollment may have led to nondifferential exposure misclassification in this study, especially for the latter half of follow-up, or the association observed early in follow-up could have been due to outliers in this period, both of which could explain why the association is less strong later compared with earlier in follow-up.

Although the mechanism through which coumaphos exposure may affect prostate cancer risk is not known at this time, OP pesticides have been shown to induce oxidative stress (Bagchi et al. 1995), which if not repaired may lead to cellular damage and disruption of DNA repair mechanisms (Muniz et al. 2008). In addition, limited experimental research suggests that OP pesticides may influence sex steroid hormone homeostasis (Gore 2002; Kang et al. 2004; Svechnikov et al. 2005; Tamura et al. 2001; Usmani et al. 2003, 2006), altering the level of circulating and/or bioavailable sex steroid hormones. This may affect cell proliferation or other important functions ultimately affecting prostate cancer risk (Hsing et al. 2002; Platz and Giovannucci 2004). Although circulating levels of steroid hormone have not been significantly associated with prostate cancer (Endogenous Hormones and Prostate Cancer Collaborative Group et al. 2008), some question the degree to which circulating hormone concentration reflects intraprostatic concentrations, the exposure of interest, and encourage continued research in this area (Hsing et al. 2008). Therefore, variation in genes involved in DNA repair or hormone synthesis and regulation, among other pathways, may help explain the observed association between coumaphos use and prostate cancer among men with a family history of disease.

Strengths of the AHS include the prospective nature of the study, high participation rates, and low loss to cancer follow-up. Given the extensive exposure information collected through the AHS, we were able to control for exposure to other pesticides as well as other occupational and lifestyle factors. Some limitations should also be noted. The small percentage of applicators reporting ever use of coumaphos (7.7%) disallowed investigation of associations between coumaphos use and many cancer sites for which too few coumaphos-exposed cancer cases occurred for a robust statistical analysis. Research in another prospective cohort study reveals that self-reported family history of prostate cancer among men is moderately reliable. Comparing self-reported family history of cancer in the Prostate, Lung, Colorectal, and Ovarian (PLCO) cohort study with Surveillance, Epidemiology and End Results (SEER) Program results indicates that men self-report 52% (95% CI, 50–55) of the expected number of prostate cancers among their first-degree relatives (Pinsky et al. 2003). Although in general, the AHS cohort has been found to have provided valid (Hoppin et al. 2002) and reliable (Blair et al. 2002) estimates of exposure, inaccurate recall is undoubtedly a limitation and would introduce random exposure misclassification, which would attenuate the RR (Greenland 1980).

We observed no association with cancer overall or any of the specific cancers investigated. We observed an increased risk of prostate cancer in association with coumaphos exposure among men with a family history of prostate cancer but not among men without a family history in both early (1993–1999) and later periods in cohort follow-up (2000–2005), although the interaction RR in the early period is stronger than in the later period. This finding suggests further monitoring of the cohort is warranted. Possible genetic susceptibility to prostate cancer associated with coumaphos exposure is suggested and should be investigated; however, because the magnitude of the association decreased over time, alternative explanations, including chance, need to be considered.

## Figures and Tables

**Table 1:** Characteristics of male AHS farmers and commercial applicators by coumaphos cumulative exposure category, 1993–2005 [no. (%)].

	Coumaphos exposure		
Characteristic	None	Low^a^	High^b^
Total	44,133 (92.3)	1,526 (3.2)	2,163 (4.5)
Age at enrollment (years)			
< 40	16,015 (36.3)	439 (28.8)	633 (29.3)
40–49	12,240 (27.7)	437 (28.6)	709 (32.8)
50–59	8,703 (19.7)	362 (23.7)	480 (22.2)
≥ 60	7,175 (16.3)	288 (18.9)	341 (15.8)
Mean age (years)	45.8	47.8	47.3
State of residence			
Iowa	30,113 (68.2)	1,071 (70.2)	1,500 (69.4)
North Carolina	14,020 (31.8)	455 (29.8)	663 (30.7)
Type of applicator			
Private	40,008 (90.7)	1,491 (97.7)	2,065 (95.5)
Commercial	4,125 (9.4)	35 (2.3)	98 (4.5)
Smoking history			
None	23,298 (53.4)	882 (58.1)	1,206 (56.2)
Former	12,904 (29.6)	443 (29.2)	670 (31.2)
Current	7,426 (17.0)	193 (12.7)	270 (12.6)
Alcohol consumption			
Never in last year	13,443 (30.9)	477 (31.4)	598 (27.9)
Ever in last year	30,099 (69.1)	1,042 (68.6)	1,548 (72.1)
Education			
High school or less	24,442 (55.4)	724 (47.4)	1,054 (48.7)
Greater than high school	19,691 (44.6)	802 (52.6)	1,109 (51.3)
Family history of any cancer			
No	25,286 (60.6)	769 (53.0)	1,103 (53.7)
Yes	16,433 (39.4)	681 (47.0)	950 (46.3)
Race			
White	40,984 (96.9)	1,435 (98.0)	2,029 (97.8)
Nonwhite	1,299 (3.1)	29 (2.0)	46 (2.2)
Fruit/vegetable intake (servings per week)			
2 or fewer per week	8,507 (19.6)	183 (12.1)	250 (11.7)
3–6 per week	17,390 (40.1)	559 (37.1)	809 (37.9)
≥ 7 per week	17,481 (40.3)	765 (50.8)	1,074 (50.4)
Cattle farmer			
No	27,806 (63.0)	383 (25.1)	460 (21.3)
Yes	16,327 (37.0)	1,143 (74.9)	1,703 (78.7)
Years of follow-up (mean ± SD)	10.0 ± 2.1	10.1 ± 2.1	10.1 ± 2.1
Lifetime no. of days of all pesticide application (mean ± SD)	379.9 ± 604.0	381.3 ± 527.2	493.1 ± 683.0

Numbers may not sum to total because of missing data; percents may not sum to 100 because of rounding. Sample is restricted to men without previous cancer diagnosis with follow-up through 2005.

a Includes men in the first tertile of cumulative exposure days (1–8.74 days).

b Includes men in the top two tertiles of cumulative exposure days (≥ 8.75 days).

**Table 2:** RRs for total and major cancers by coumaphos lifetime exposure days among males, AHS 1993–2005.

Cancer site	Cumulative coumaphos exposure days	Cancer cases (no.)^a^	Person-years	Nonexposed referent^b^,^c^ RR (95% CI)	Low-exposed referent^b^,^c^ RR (95% CI)
All cancer	None	2,960	17,260	1.00	—
	1.0–8.74	114	655	1.00 (0.82–1.23)	1.00
	8.75–38.75	71	392	0.92 (0.71–1.20)	0.92 (0.67–1.28)
	> 38.75	73	397	0.96 (0.74–1.25)	0.96 (0.69–1.33)
	*p* for trend			0.70	0.76

Prostate	None	1,196	6,860	1.00	—
	1.0–8.74	57	346	1.17 (0.88–1.56)	1.00
	8.75–38.75	28	145	0.84 (0.60–1.26)	0.72 (0.44–1.17)
	> 38.75	30	187	0.93 (0.62–1.41)	0.80 (0.48–1.31)
	*p* for trend			0.68	0.34

Lung	None	288	1,725	1.00	—
	1.0–8.74	10	48	1.24 (0.64–2.42)	1.00
	8.75–38.8	5	26	1.03 (0.43–2.52)	0.84 (0.28–2.50)
	> 38.8	7	42	0.78 (0.29–2.11)	0.63 (0.19–2.05)
	*p* for trend			0.67	0.52

Colorectal	None	342	2,076	1.00	—
	1.0–8.74	12	71	0.80 (0.41–1.55)	1.00
	8.75–38.8	5	25	0.52 (0.19–1.40)	0.65 (0.20–2.13)
	> 38.8	6	22	0.84 (0.37–1.89)	1.06 (0.38–2.97)
	*p* for trend			0.49	0.65

Lymphohematopoietic	None	295	1,740	1.00	—
	1.0–8.74	11	54	0.86 (0.44–1.68)	1.00
	8.75–38.8	7	45	0.85 (0.38–1.91)	0.99 (0.35–2.77)
	> 38.8	7	31	1.08 (0.51–2.30)	1.26 (0.47–3.38)
	*p* for trend			0.91	0.79

a Cancer registry follow-up through 2005.

b Adjusted for age, education, state, smoking, family history, fruit and vegetable consumption, total days of application of any pesticide.

c The nonexposed are those who reported never using coumaphos; the low-exposed referents are those in the low-exposure tertile.

**Table 3:** Association between ever use of coumaphos and prostate cancer RRs (95% CI) by family history of prostate cancer, early (1993–1999) and later (2000–2005) in cohort follow-up, AHS.

	Family history of prostate cancer						
	No^a^			Yes^a^			Interaction
Time period	Cases	P-Y	RR^b^ (95% CI)	Cases	P-Y	RR^b^ (95% CI)	RR^c^ (95% CI)
Early (1993–1999)^d^							
Never	353	889	1.00	71	200	1.00	—
Ever	25	56	0.78 (0.52–1.17)	15	41	2.07 (1.19–3.62)	2.67 (1.34–5.31)
*p* for interaction							0.005

Late (2000–2005)							
Never	582	4,520	1.00	113	859	1.00	—
Ever	50	403	0.93 (0.69–1.23)	18	144	1.46 (0.89–2.40)	1.60 (0.90–2.82)
*p* for interaction							0.113

Entire period							
Never	935	5,409	1.00	184	1,059	1.00	—
Ever	75	459	0.87 (0.68–1.10)	33	185	1.65 (1.13–2.38)	1.91 (1.23–2.95)
*p* for interaction							0.004

P-Y, person-years. Using the lifetime exposure days metric we examined prostate cancer risk by coumaphos exposure response patterns. The number of exposed cases was small, making the following risk estimates unstable: none, low (> 8.75 days), high (> 8.75 days), RR without prostate cancer family history 1.0, 0.98 (0.71–1.35), and 0.78 (0.56–1.08), *p*-trend 0.15, and with a family history, 1.0, 2.06 (1.25–3.38), and 1.36 (0.81–2.26), *p*-trend 0.04, *p*-interaction 0.009.

a Seven coumaphos-exposed prostate cancer cases were missing information on family history.

b Adjusted for age.

c Adjusted for age and family history of prostate cancer. The interaction RR is the exponentiation of the interaction coefficient and can be interpreted as the ratio of the joint effect of both exposures versus the expected joint effect of each exposure singly, assuming a multiplicative model.

d Previously reported by Alavanja et al. (2003).
